# Expanding the perception of medical students on prehospital care through supervised observation

**DOI:** 10.1111/medu.15650

**Published:** 2025-03-10

**Authors:** Elena Ahjem, Lisa Ramage, Yousef Maait

## 1 WHAT PROBLEMS WERE ADDRESSED?

UK medical students have little opportunity to gain experience in pre‐hospital emergency medicine (PHEM). Before this programme, students at Cambridge University were not offered a chance to observe how healthcare is delivered without hospital resources or understand the experiences of patients in out‐of‐hospital scenarios. Thus, the curriculum overlooked the benefits that PHEM experience offers. For example, students express a lack of confidence in approaching acutely ill patients or managing trauma situations.^1^ Thus, PHEM can diversify students' learning environments and give them exposure to patients whom they would otherwise miss and, therefore, help to develop their confidence.

## 2 WHAT WAS TRIED?

The Cambridge Pre‐hospital Care Programme (PCP) aimed to establish pre‐hospital care as an option for interested students by integrating it into the curriculum as a student‐selected component (SSC). 1 In partnership with the University of Cambridge and local PHEM services, a programme was designed and piloted in the 2023/2024 academic year, seeing four year‐4 clinical students take part. During the year‐long programme, students undertook research projects (supervised by a mentor) and attended shifts with local services including the East of England Ambulance Service Trust, Essex & Herts Air Ambulance Trust, MAGPAS Air Ambulance and BASICS Essex. Students were provided with learning objectives and a list of clinical skills that they were to fulfil, experience or discuss during their shifts, and they were also encouraged to develop their reflective practice by completing reports on each patient they attended. This interrogative approach was promoted further by regular debriefing sessions. Students were expected to attend monthly PCP forums where experts were invited to deliver a range of lectures, and by the end of the year, students presented their projects and a clinical case at one of these forums.

## 3 WHAT LESSONS WERE LEARNED?

In the programme feedback, students reported getting a better understanding of how to handle stress, apply their clinical knowledge and effectively communicate with patients and their families. Feedback identified that students benefitted from developing interprofessional relationships among the PHEM multi‐disciplinary team and networking with senior colleagues within the speciality who provided guidance. Students found particular benefit in witnessing clinicians being adaptable in their decision‐making as patients' conditions evolved. The interprofessional collaboration was reciprocal because an environment was created where insights into the roles performed by each profession within the wider picture of our healthcare service were shared. For students, the programme puts into perspective the patients' journey of care before arriving in the hospital, the role of various healthcare providers and the wider team and helps to contextualise the pre‐hospital to in‐hospital transitional phase of care.

Some challenges were encountered in the set‐up and delivery of the programme, predominantly centred around the organisation of shifts across services, which required liaising with each service individually. Additionally, there was some competition for shifts with other observers from different backgrounds, including paramedic students on university placement and elective students. Despite this, the PCP students showed commitment to the programme and were adaptable, so that all programme objectives were addressed fully.

## AUTHOR CONTRIBUTIONS

**Elena Ahjem:** Writing—original draft; writing—review and editing. **Lisa Ramage:** Writing—review and editing; conceptualization; supervision. **Yousef Maait:** Supervision; writing—review and editing.

## Data Availability

Data sharing is not applicable to this article as no new data were created or analysed in this study.

## Footnotes

^1^
We would like to extend our gratitude to the University of Cambridge Clinical School and Dr Nicholas Evans for their kind collaboration with the programme. 
